# ^18^F-FDG PET/CT Scans Can Identify Sub-Groups of NSCLC Patients with High Glucose Uptake in the Majority of Their Tumor Lesions

**DOI:** 10.7150/jca.45899

**Published:** 2021-01-01

**Authors:** Anne M. Hendriks, Adrienne H. Brouwers, Panagiotis Giannopoulos, Joop D. Lefrandt, Wim Timens, Harry J.M. Groen, Geertruida H. de Bock, Mathilde Jalving

**Affiliations:** 1 University Medical Center Groningen, University of Groningen, Hanzeplein 1, 9713 GZ, Groningen, the Netherlands. Department of Medical Oncology.; 2 University Medical Center Groningen, University of Groningen, Hanzeplein 1, 9713 GZ, Groningen, the Netherlands. Department of Nuclear Medicine and Molecular Imaging.; 3 University Medical Center Groningen, University of Groningen, Hanzeplein 1, 9713 GZ, Groningen, the Netherlands. Department of Internal Medicine.; 4 University Medical Center Groningen, University of Groningen, Hanzeplein 1, 9713 GZ, Groningen, the Netherlands. Department of Pathology.; 5 University Medical Center Groningen, University of Groningen, Hanzeplein 1, 9713 GZ, Groningen, the Netherlands. Department of Pulmonary Diseases.; 6 University Medical Center Groningen, University of Groningen, Hanzeplein 1, 9713 GZ, Groningen, the Netherlands. Department of Epidemiology.

**Keywords:** ^18^F-FDG PET/CT, non-small cell lung cancer, glycolysis, type 2 diabetes mellitus

## Abstract

**Background:** Reprogrammed glucose metabolism is a hallmark of cancer making it an attractive therapeutic target, especially in cancers with high glucose uptake such as non-small cell lung cancer (NSCLC). Tools to select patients with high glucose uptake in the majority of tumor lesions are essential in the development of anti-cancer drugs targeting glucose metabolism. Type 2 diabetes mellitus (T2DM) patients may have tumors highly dependent on glucose uptake. Surprisingly, this has not been systematically studied. Therefore, we aimed to determine which patient and tumor characteristics, including concurrent T2DM, are related to high glucose uptake in the majority of tumor lesions in NSCLC patients as measured by 2-deoxy-2-[fluorine-18]fluoro-D-glucose (^18^F-FDG) positron emission tomography (PET)/computed tomography (CT) scans.

**Methods:** Routine primary diagnostic ^18^F-FDG PET/CT scans of consecutive NSCLC patients were included. Mean standardized uptake value (SUVmean) of ^18^F-FDG was determined for all evaluable tumor lesions and corrected for serum glucose levels according to the European Association of Nuclear Medicine Research Ltd guidelines. Patient characteristics potentially determining degree of tumor lesion glucose uptake in the majority of tumor lesions per patient were investigated.

**Results:** The cohort consisted of 102 patients, 28 with T2DM and 74 without T2DM. The median SUVmean per patient ranged from 0.8 to 35.2 (median 4.2). T2DM patients had higher median glucose uptake in individual tumor lesions and per patient compared to non-diabetic NSCLC patients (SUVmean 4.3 vs 2.8, *P* < 0.001 and SUVmean 5.4 vs 3.7, *P* = 0.009, respectively). However, in multivariable analysis, high tumor lesion glucose uptake was only independently determined by number of tumor lesions ≥1 mL per patient (odds ratio 0.8, 95% confidence interval 0.7-0.9).

**Conclusions:** ^18^F-FDG PET/CT scans can identify sub-groups of NSCLC patients with high glucose uptake in the majority of their tumor lesions. T2DM patients had higher tumor lesion glucose uptake than non-diabetic patients. However, this was not independent of other factors such as the histological subtype and number of tumor lesions per patient.

## Introduction

Reprogrammed energy metabolism is a hallmark of cancer, of which the most well-known example is the so-called Warburg effect or aerobic glycolysis describing high glycolysis rates even in the presence of sufficient oxygen and functional mitochondria 1. This phenomenon is essential for production of biomass and maintaining redox balance which benefits cancer cell growth and division 1. Therefore, glycolysis is a potentially attractive therapeutic target, particularly in tumor types with especially high glucose uptake such as non-small cell lung cancer (NSCLC). Targeting glycolysis can, at least in preclinical models, reduce cancer cell growth and enhance effectiveness of chemotherapy, immunotherapy and radiation therapy 2,3. Drugs targeting various glycolytic enzymes are in clinical development, however, no clear efficacy signals have emerged and toxicity is a problem at higher doses 4. Clinical trials have, so far, not been enriched for patients with tumors highly dependent on glucose uptake and have excluded patients with concurrent type 2 diabetes mellitus (T2DM) 5-11. T2DM is characterized by insulin resistance of liver, muscle and fat tissue resulting in hyperinsulinemia and hyperglycemia, which are both associated with increased cancer risk and cancer-related mortality 12,13. Insulin resistance does not occur in epithelial cells, of which many types of cancer are derived, resulting in relatively high insulin and glucose exposure of cancer cells in T2DM patients 12,13. Therefore, patients with T2DM may be especially likely to have tumors highly dependent on glycolysis.

There is an unmet need for methods to enrich study populations for patients with highly glucose dependent tumors. An obvious candidate, which has not been explored for use as a predictive biomarker in this manner, is the 2-deoxy-2-[fluorine-18]fluoro-D-glucose (^18^F-FDG) positron emission tomography (PET)/computed tomography (CT) scan. ^18^F-FDG is phosphorylated to ^18^F-FDG-6-phosphate but cannot be further metabolized and therefore accumulates in cells. In this way, ^18^F accumulation reflects the amount of glucose entering the cell and glucose phosphorylation, and can therefore be considered as an indirect measure of the rate of glycolysis.

^18^F-FDG PET/CT scans are part of the routine diagnostic work-up in NSCLC 14-16. In NSCLC patients, high ^18^F-FDG uptake rates correlate with increased immunohistochemical expression of the glucose transporter 1 (GLUT-1) and upregulation of the rate-determining glycolytic enzyme hexokinase 1 17. Furthermore, high tumor lesion glucose uptake as measured using ^18^F-FDG-based parameters, such as tumor lesion ^18^F-FDG uptake and metabolic tumor volume (MTV), are associated with worse prognosis in NSCLC patients 17-19. Patients with NSCLC have an increased risk of concurrent T2DM due to overlapping risk factors of these two diseases 20-22. Therefore, in NSCLC patients, we retrospectively studied whether ^18^F-FDG PET/CT scans can be used to identify patients with high ^18^F-FDG uptake in the majority of tumor lesions. Furthermore, we used ^18^F-FDG PET/CT scans to investigate whether specific characteristics of NSCLC patients, including concurrent T2DM, relate to high glucose uptake in the majority of tumor lesions per patient.

## Methods

### Study design and patient selection

This was a cohort study including a consecutive series of patients diagnosed with NSCLC. The main outcome was^ 18^F-FDG tumor lesion uptake. The main variables considered were diabetic state, age, sex, stage of disease, histological subtypes, number of tumor lesions and MTV.

**Figure 1:**
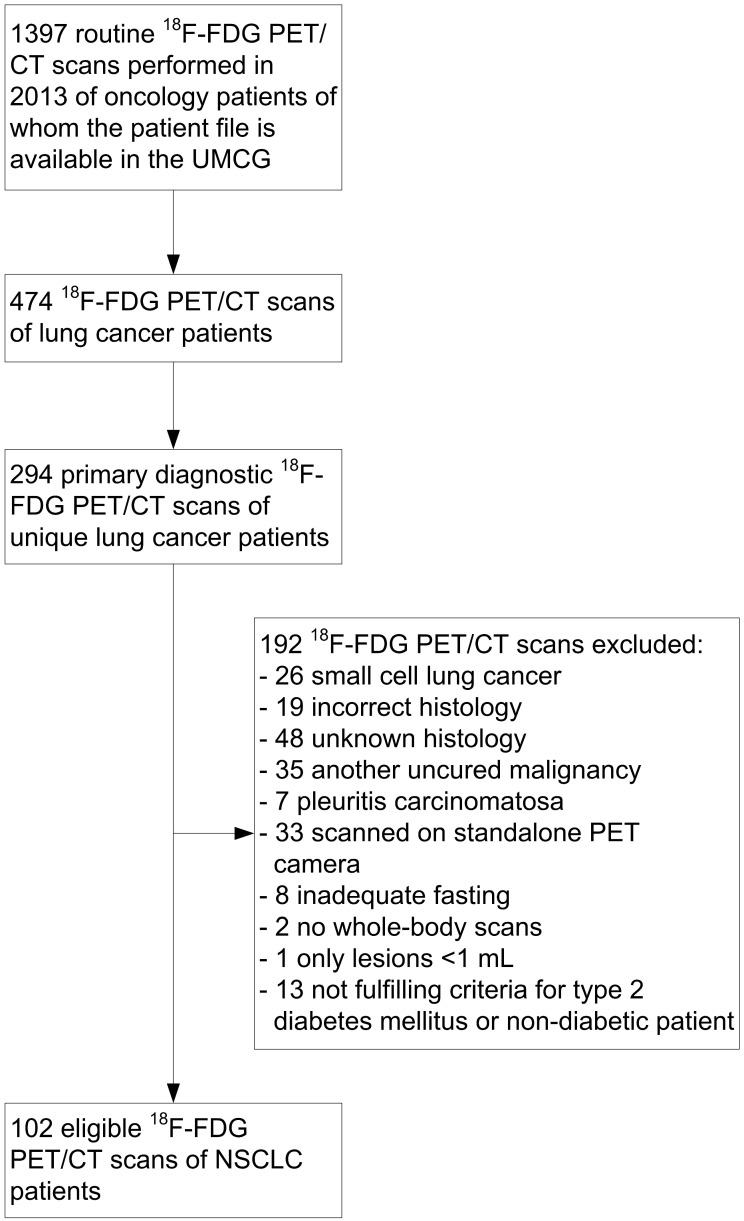
** CONSORT diagram of 2-deoxy-2-[fluorine-18]fluoro-D-glucose (^18^F-FDG) positron emission tomography (PET)/computed tomography (CT) scans in non-small cell lung cancer (NSCLC) patients.** UMCG: University Medical Center Groningen.

All ^18^F-FDG PET/CT scans performed at the University Medical Center Groningen in NSCLC patients in 2013 were selected (**Fig. 1**). The routine primary diagnostic scans of these patients were identified and included. Primary diagnostic ^18^F-FDG PET/CT scans were defined as scans used for the pre-treatment diagnosis of NSCLC or for the detection of recurrence at least 1 year after the last anti-cancer therapy. Only ^18^F-FDG PET/CT scans of patients with a pathologically confirmed diagnosis of NSCLC with adenocarcinoma, squamous cell carcinoma, adenosquamous carcinoma or large cell carcinoma histology were included. Patients diagnosed with another uncured malignancy were excluded. Patients with pleuritis carcinomatosa or only low-volume tumor lesions (<1 mL) were also excluded, since accurate tumor delineation on a PET image is not possible in these cases.

Patients previously diagnosed with T2DM, on oral anti-diabetic treatment or with fasting serum glucose levels ≥7.0 mmol/L were defined as T2DM patients 23. Patients with a fasting serum glucose level ≤6.4 mmol/L, not previously diagnosed with diabetes and not on any diabetes medication were defined as non-diabetic. The cohort did not contain type 1 diabetes mellitus (T1DM) patients.

Data obtained from patient records and scans were anonymously stored using study-specific patient codes in a password-protected database. Institutional review board approval for this study was obtained and the need for informed consent was waived (Medical Ethical Committee number: 2018/508).

### PET/CT imaging technique

^18^F-FDG PET/CT scanning was performed according to the European Association of Nuclear Medicine Research Ltd (EARL) guidelines version 1.0 24 and additional local protocols, most of which have become standard of care with the EARL guidelines version 2.0 25. ^18^F-FDG PET/CT scans were performed using a mCT scanner (Siemens/CTI, Knoxville, TN). A protocol with 3 dimensional mode, 1-3 min emission time per bed position dependent on patient weight, 2 mm spatial resolution and a non-contrast enhanced low dose CT scan for attenuation correction were used. Reconstruction was performed using a Gaussian filter of 6.5 mm in full width at half maximum and iterative reconstruction methods with 3 iterations and 21 subsets.

The non-diabetic patients fasted for at least 6 h before intravenous administration of ^18^F-FDG. Known insulin-independent T2DM patients complied with the standard fasting protocol and continued oral anti-diabetic drugs. Known insulin-dependent T2DM patients had a meal and their normal insulin dose at least 4 h before ^18^F-FDG administration and then fasted until the end of the procedure.

Before injecting ^18^F-FDG, the fasting serum glucose level was measured by calibrated venous blood sampling (Accu-Chek Inform II, Roche, Basel, Switzerland). These fasting serum glucose levels and the patient records were available for all patients. Scans were rescheduled in cases when the patient's fasting serum glucose level was >11 mmol/L. Patients were injected with 3 MBq/kg ^18^F-FDG 60 min before scanning.

### ^18^F-FDG uptake measurements

A nuclear medicine physician assessed all scans for routine care at the time they were performed. All ^18^F-FDG avid tumor lesions were reassessed by three investigators (AMH, AHB, PG). A region of interest (ROI) was drawn around each visible ^18^F-FDG avid tumor lesion. In case of doubt whether an ^18^F-FDG avid tumor lesion was malignant, the images were reviewed together with the corresponding CT images. Based on the threshold method, the tumor lesion's ^18^F-FDG uptake was determined based on all voxels with an uptake higher than 40% of the maximum measured standardized uptake value (SUVmax) in the ROI 26. The mean of all SUVs measured in these voxels (SUVmean) was used as the parameter for tumor lesion glucose uptake per ROI. SUVmean was chosen since it represents the overall uptake value in the measured area, instead of SUVmax which represents just one voxel 27. SUVmean values were corrected for patients' serum glucose levels according to the EARL guidelines (SUVcorrected = SUVmeasured × (fasting serum glucose level (mmol/L) / 5)) 24,25. These corrected SUVmean values were used for all analyses unless indicated otherwise. The volume of the area on the PET image in which the SUVmean was determined was defined as the MTV 28.

The analysis of glucose uptake was only performed for tumor lesions with a MTV ≥1 mL to avoid underestimation of SUVs in smaller tumor lesions due to partial volume effects (PVE). PVE refers to factors influencing the measured amount of radioactivity within a volume of interest (*e.g.* tumor), such as the spatial resolution of the imaging system, the size and shape of the tumor lesions, and reconstruction procedures followed after acquisition of the images 29. The cut-off value of 1 mL was chosen based on the resolution of the camera used. For determining the sum of the MTV measured in all tumor lesions per patient (MTVpatient), tumor lesions with a MTV <1 mL were taken into account to minimize underestimation of the total volume. The range of all measured SUVmean values in a patient was used as a measure for intra-patient heterogeneity and was defined as the highest minus the lowest SUVmean value measured in all tumor lesions per patient. Since no studies have been performed to determine cut-off values for high ^18^F-FDG uptake using SUVmean, cut-off values were chosen based on published SUVmax data. High tumor glucose uptake was defined as a SUVmean >5 and very high glucose uptake as a SUVmean >8 30-34.

^18^F-FDG uptake in the ROIs and the associated volumes were calculated using AMIDE software, which provides raw non-smoothed data, as described in the supplemental methods.

### Statistical analysis

The cohort was described, overall and stratified by T2DM status. The Mann-Whitney U test was used to compare differences between the lowest and highest SUVmean value per patient, differences in number of tumor lesions, differences in fasting serum glucose levels and to compare median glucose uptake in individual tumor lesions and per patient among subgroups of patients. Subsequently, we evaluated the association between patient characteristics and high tumor lesion glucose uptake. For analyses including histology, only histological subtypes that occur in >5% of patients were included. Diabetic state was evaluated as the main factor. The effects of age, sex, stage of disease, histology, number of tumor lesions ≥1 mL per patient, and MTVpatient were also evaluated by univariable and multivariable logistic regression analyses using the enter method. High tumor lesion glucose uptake (SUVmean >5) in at least half of the tumor lesions per patient was used as dependent variable for these analyses to estimate odds ratios (ORs) and 95% confidence intervals (95% CI). A cut-off of at least half of the tumor lesions per patient was chosen because it is clinically relevant to be able to select patients of whom the majority of tumor lesions may be sensitive to anti-glycolytic treatment. The median MTVpatient of patients included in logistic regression analyses was used to categorize MTVpatient to determine ORs. *P* values <0.05 were considered statistically significant. IBM SPSS Statistics 23 was used for all statistical analyses.

## Results

### ^18^F-FDG tumor lesion uptake is heterogeneous in the total NSCLC cohort

**Figure 2:**
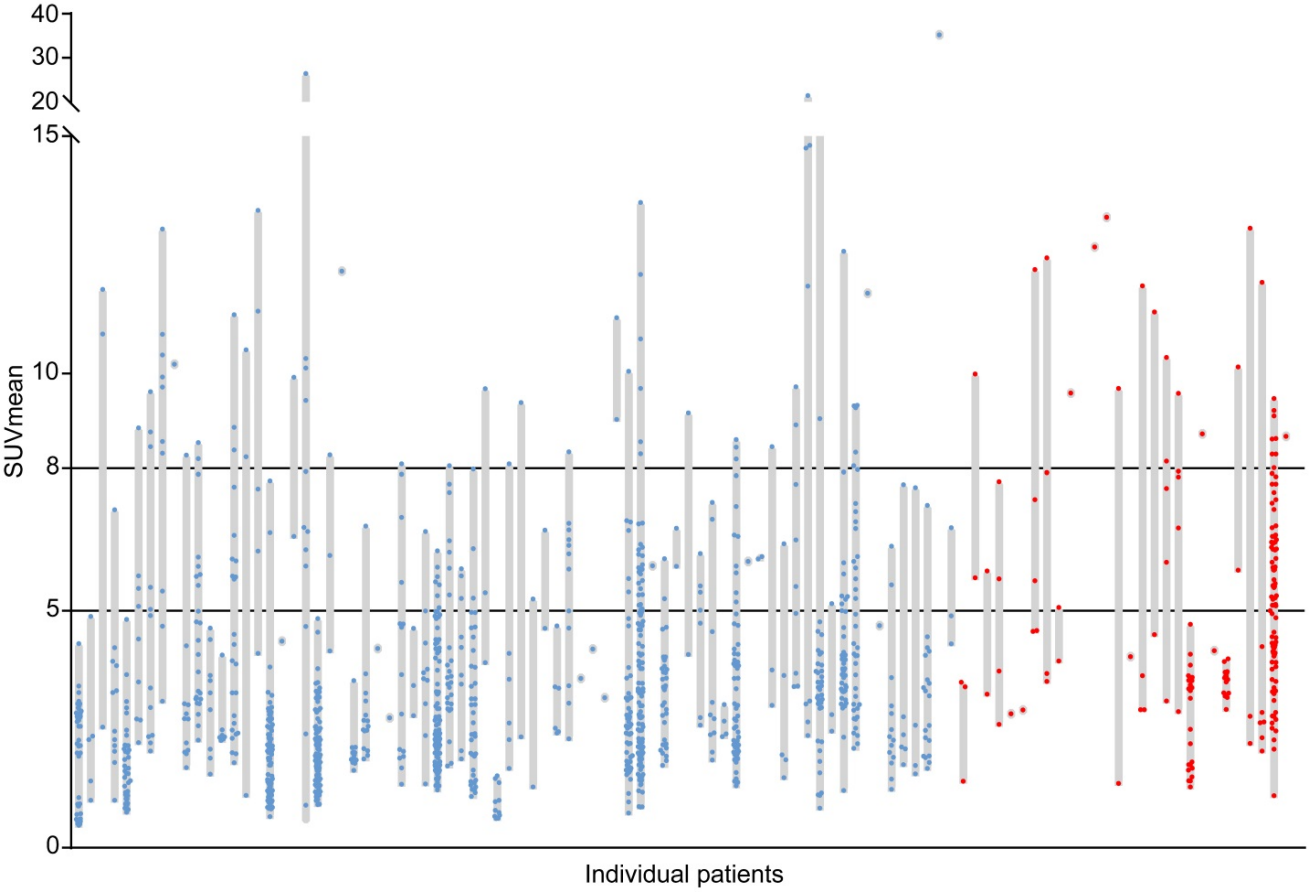
** Inter-patient and intra-patient heterogeneity in tumor lesion glucose uptake.** Each grey bar represents an individual patient. The length of the grey bars represents the range of the mean standardized uptake values (SUVmean) measured in all tumor lesions (dots) with a volume ≥1 mL visible on the primary diagnostic ^18^F-FDG PET/CT scans of the patients. Blue dots represent tumor lesions of non-diabetic patients with non-small cell lung cancer (*N*=74, *N*_lesions_=1205). Red dots represent tumor lesions of non-small cell lung cancer patients with concurrent type 2 diabetes mellitus (*N*=28, *N*_lesions_=189).

The cohort consisted of 102 NSCLC patients. **Table 1** shows the patient and tumor characteristics. The relatively high incidence of T2DM in this cohort reflects the high incidence of T2DM in the Northern Netherlands and the overlapping behavioral risk factors for T2DM and NSCLC. A large inter-patient heterogeneity in tumor lesion glucose uptake was found with median SUVmean values per patient ranging from 0.8 to 35.2 (median 4.2) (**Fig. 2**). In addition, a large intra-patient heterogeneity in tumor lesion glucose uptake was found, with differences between the lowest and highest SUVmean value per patient ranging from 0.1 to 25.5 (median 5.8) in patients with more than one tumor lesion (*N* = 79) (**Fig. 2**). In 42% of patients high (SUVmean >5) and in 22% very high (SUVmean >8) glucose uptake in at least half of the tumor lesions was found (**Fig. 2**).

### T2DM patients had higher^ 18^F-FDG tumor lesion uptake than non-diabetic patients

**Figure 3:**
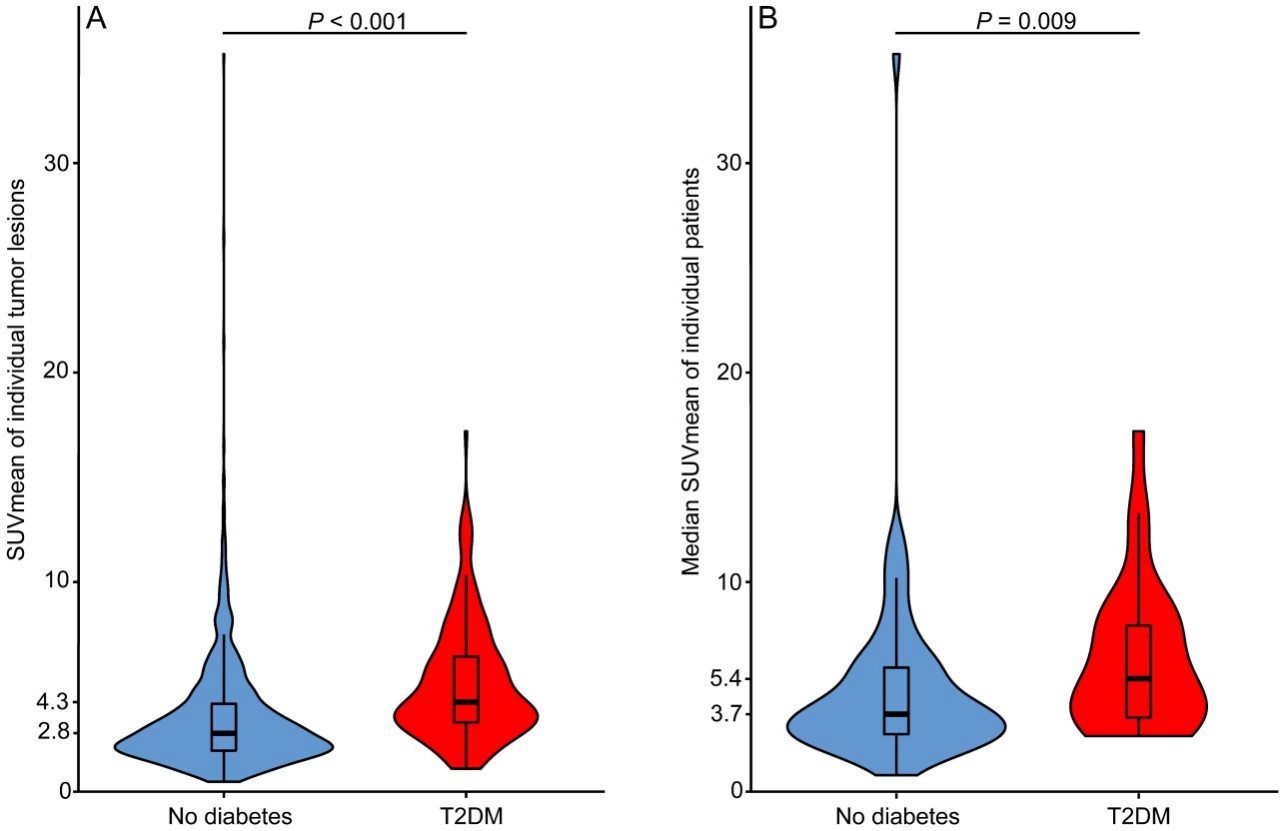
** Median tumor lesion glucose uptake is higher in type 2 diabetes mellitus (T2DM) than in non-diabetic patients. A)** Violin plot of all mean standardized uptake values (SUVmean) measured in all tumor lesions with a volume ≥1 mL visible on the primary diagnostic ^18^F-FDG PET/CT scans of non-diabetic (*N*_lesions_=1205) and type 2 diabetes mellitus (T2DM) (*N*_lesions_=189) non-small cell lung cancer (NSCLC) patients. **B)** Violin plot of the median SUVmean value per patient plotted for non-diabetic (*N*=74) and T2DM (*N*=28) NSCLC patients. Box plots showing the median (horizontal bar), the first and third quartile.

**Table 1** shows the tumor and patient characteristics stratified for T2DM and non-diabetic patients. T2DM patients had higher fasting serum glucose levels than non-diabetic NSCLC patients (*P* < 0.001). In patients with more than one tumor lesion, the difference between the lowest and highest SUVmean value per patient ranged in T2DM patients (*N* = 18) from 1.1 to 10.9 (median 6.8) and in non-diabetic patients (*N* = 61) from 0.1 to 25.5 (median 5.0) (*P* = 0.615). The median glucose uptake was higher in tumor lesions of NSCLC patients with T2DM than in tumor lesions of non-diabetic NSCLC patients (SUVmean 4.3 vs. 2.8, *P* < 0.001) (**Fig. 3A**). Median tumor lesion glucose uptake per patient was also higher for T2DM than for non-diabetic patients (SUVmean 5.4 vs 3.7, *P* = 0.009) (**Fig. 3B**). Univariable logistic regression showed that T2DM patients are more likely to have high tumor lesion glucose uptake (SUVmean >5) in at least half of the tumor lesions than non-diabetic NSCLC patients (OR 2.9, 95% CI 1.1-7.5) (**Table 2**). Six patients were on insulin treatment, the distribution of ^18^F-FDG tumor lesion uptake in these patients did not differ from the distribution in the T2DM patients not on insulin treatment (**Fig. S1**).

### T2DM is not the main determinant of increased ^18^F-FDG uptake in the majority of tumor lesions

To determine whether T2DM was independently associated with high tumor lesion glucose uptake, univariable logistic regression was used to determine the OR of other potentially relevant patient characteristics on high tumor lesion glucose uptake. Squamous cell carcinoma histology (OR 4.0, 95% CI 1.7-9.5) and low number of tumor lesions ≥1 mL per patient (OR 0.8, 95% CI 0.7-0.9) were shown to be also associated with high tumor lesion glucose uptake (SUVmean >5) in at least half of the tumor lesions (**Table 2**). The variables that were significantly associated with high tumor lesion glucose uptake in univariable analyses were included in multivariable logistic regression analysis. Based on this analysis, low number of tumor lesions ≥1 mL per patient was shown to be independently associated with high ^18^F-FDG uptake in the majority of tumor lesions (OR 0.8, 95% CI 0.7-0.9) (**Table 2**).

## Discussion

In this study cohort, a large inter-patient and intra-patient heterogeneity in tumor lesion glucose uptake was found in NSCLC patients. ^18^F-FDG PET/CT scans could identify sub-groups of NSCLC patients with high (SUVmean >5) glucose uptake in the majority (≥ 50%) of their tumor lesions. T2DM NSCLC patients had higher median glucose uptake in individual tumor lesions and per patient compared to non-diabetic NSCLC patients. However, this was not independent of other factors such as the histological subtype and number of tumor lesions per patient.

The relationship between mean ^18^F-FDG tumor lesion uptake and concurrent T2DM has not previously been systematically studied in well-selected metastatic lung cancer patients with and without T2DM. In other studies comparing diabetic and non-diabetic cancer patients, ^18^F-FDG PET/CT scans were not performed according to EARL standards for glucose correction, performed at unclear time points during anti-tumor therapy and used SUVmax or SUVpeak instead of SUVmean to determine tumor lesion ^18^F-FDG uptake 35-40. In addition, four of these studies only measured ^18^F-FDG uptake in one tumor lesion per patient and only one study clearly defined whether only T2DM or also T1DM patients were included 35-40. Two of the previous studies were conducted in lung cancer patients, one reporting reduced tumor lesion glucose uptake in diabetic patients 35 and one reporting no difference between diabetic and non-diabetic patients 37. However, both of these studies did not perform multivariable analysis, one of these studies included only five diabetic patients and various histological subtypes including small cell lung cancer 35, whereas the other study did not specify which histological subtypes were included 37. The other four previous studies were conducted in various cancer types reporting both reduced and equal tumor lesion glucose uptake in diabetic compared to non-diabetic patients 36,38-40. *In vitro* data support the hypothesis that tumor metabolism differs between T2DM and non-diabetic patients. Hyperglycemia and hyperinsulinemia, both characteristics of T2DM, have been shown to promote cancer cell proliferation, survival and migration in preclinical models 12,13. Expression of insulin-like growth factor 1 receptor, which stimulates glucose uptake, is higher in NSCLC tumors from diabetic patients than in tumors from non-diabetic patients 41. Evidence exists that increased cellular glucose uptake may increase the sensitivity of drugs targeting glycolysis. Stimulation of glucose uptake in colorectal cancer cell lines by insulin enhanced cytotoxicity induced by the glycolysis inhibitor 2-deoxyglucose (2-DG) 42. Furthermore, 2-DG treatment enhanced effects of radiation therapy in highly versus normally glycolytic cancer cell lines 43.

T2DM, squamous cell carcinoma histology and low number of tumor lesions ≥1 mL per patient were associated with high tumor lesion glucose uptake in the majority of tumor lesions per patient in univariable analyses. However, this was not the case in multivariable analysis where only low number of tumor lesions remained significant. Since patients with a higher number of tumor lesions had a higher frequency of smaller tumor lesions (**Figure S2**), the association between number of tumor lesions and ^18^F-FDG tumor lesion uptake may be explained by PVE. In smaller lesions there is greater risk of SUV underestimation due to PVE 29. In a phantom study according to EARL guidelines and the delineation method we used, SUVmean values are underestimated by at least 40% in a sphere of 1.15 mL, 27% in a sphere of 2.57 mL and 11% in a sphere of 26.53 mL 44,45. The median number of tumor lesions was lower in T2DM than in non-diabetic patients and also lower in patients with squamous cell carcinoma than in those with adenocarcinoma histology (**Figure S3**). The lower number of tumor lesions in T2DM patients may be explained by detection bias 46. Regular T2DM-related medical check-ups could lead to diagnosis of cancer at an earlier time point with lower numbers of tumor lesions, as has been previously reported for colorectal cancer 47. Higher tumor lesion glucose uptake in squamous cell carcinoma as compared to adenocarcinoma histology has been previously reported and may be caused by high GLUT-1 expression 30,48-50. More frequent occurrence of squamous cell carcinoma compared to adenocarcinoma histology in T2DM compared to non-diabetic NSCLC patients, as we demonstrated, has not been described previously. This result is in line with data showing that tobacco smokers with NSCLC are more likely to have squamous histology, and that life-style habits of smokers, such as dietary habits, put them at risk for T2DM 21,51.

We show a large inter-patient and intra-patient heterogeneity in tumor lesion glucose uptake in NSCLC patients measured as SUVmean, representing the average amount of ^18^F-FDG uptake in a tumor lesion. Previous smaller studies have only investigated the maximum ^18^F-FDG uptake measured in NSCLC tumor lesions, which gives a less accurate estimate of the actual glucose uptake in the entire tumor lesion 30,52-54. Furthermore, these studies did not show how large the variation in tumor lesion glucose uptake can be within a single patient 30,52-54. At this time no validated predictive biomarkers for patient selection for drugs targeting glycolysis are available. A potential tool for such purpose is using ^18^F-FDG PET/CT scans as described in this study, to identify patients with high glucose uptake defined as SUVmean >5 in the majority (≥50%) of tumor lesions. Prospective clinical studies testing drugs targeting glycolysis, such as 2-DG, may consider incorporation of baseline ^18^F-FDG PET/CT scans to investigate suitability as a potential predictive biomarker.

A limitation of the current study is that no data was available regarding treatment of T2DM patients with oral blood glucose lowering agents due to the retrospective design. Metformin, the most prescribed oral anti-diabetic drug, is known to increase bowel ^18^F-FDG uptake 55, but it is not yet understood whether metformin influences tumor ^18^F-FDG uptake 56,57. A small prospective randomized controlled trial showed no influence of metformin on ^18^F-FDG uptake in tumors, liver, heart, bone marrow and skeletal muscle 55. In accordance with previous retrospective studies 39,58, we demonstrated that insulin treatment of T2DM patients did not influence the distribution of ^18^F-FDG uptake in NSCLC tumor lesions. Missed tumor lesions during assessment of the ^18^F-FDG PET/CT scans is unlikely since sub-types of NSCLC known for low ^18^F-FDG uptake, such as bronchioloalveolar carcinoma 59, were excluded during patient selection.

In conclusion, a large inter-patient and intra-patient heterogeneity in tumor lesion glucose uptake was found in NSCLC patients. ^18^F-FDG PET/CT scans could identify sub-groups of NSCLC patients with high glucose uptake (SUV mean >5) in the majority (≥ 50%) of their tumor lesions. T2DM patients had higher median glucose uptake in individual tumor lesions and per patient than non-diabetic NSCLC patients. Although this was not independent of other factors such as the histological subtype and number of tumor lesions per patient, our data may suggest that tumors from T2DM NSCLC patients have a different biology than tumors from non-diabetic NSCLC patients. A prospective study is required to elucidate the underlying biology and determine the potential for ^18^F-FDG PET/CT as a tool to select patients for metabolically targeted anti-cancer therapies.

## Supplementary Material

Supplementary figures.

## Figures and Tables

**Table 1:** Tumor and patient characteristics of the total non-small cell lung cancer (NSCLC) patients' cohort and stratified for the non-diabetic and type 2 diabetes mellitus (T2DM) patients

Characteristics	All patients	Non-diabetic	T2DM
Patients, *N*	102	74	28
Age in years, median (range)	65 (32-89)	63 (32-89)	67 (48-80)
Male, N (%)	58 (57%)	35 (47%)	23 (82%)
Fasting serum glucose level (mmol/L), median (range)	5.6 (3.1-10.2)	5.3 (3.9-6.4)	7.3 (3.1-10.2)
**Stage of disease, N (%)**			
1	15 (15%)	8 (11%)	7 (25%)
2	7 (7%)	5 (7%)	2 (7%)
3	34 (33%)	19 (25%)	15 (54%)
4	46 (45%)	42 (57%)	4 (14%)
**Histology, N (%)**			
Adenocarcinoma	60 (59%)	49 (66%)	11 (39%)
Squamous cell carcinoma	36 (35%)	22 (30%)	14 (50%)
Adenosquamous carcinoma	3 (3%)	1 (1%)	2 (7%)
Large cell carcinoma	3 (3%)	2 (3%)	1 (4%)
**Tumor lesions per patient, median (range)**			
All tumor lesions	4.5 (1-145)	7 (1-145)	2 (1-94)
Tumor lesions ≥ 1 mL	4 (1-116)	6.5 (1-116)	2 (1-89)
**Total tumor lesions, N**			
All tumor lesions	1550	1354	196
Tumors ≥ 1 mL	1394	1205	189
MTV per tumor lesion (mL), median (range)	2.9 (1.0-588.0)	2.8 (1.0-588.0)	4.4 (1.1-203.4)
MTV per patient (mL), median (range)	72.3 (1.7-1666.4)	79.8 (1.7-1666.4)	34.6 (2.0-743.6)

MTV, metabolic tumor volume; MTV per patient is the sum of the MTV measured in all tumor lesions per patient; *N,* number.

**Table 2:** Univariable and multivariable associations between patient characteristics and high tumor lesion glucose uptake (SUVmean >5) in at least half of the tumor lesions per patient

Variable	Reference variable	Univariable analysis		Multivariable analysis	
		OR (95% CI)	*P*-value	OR (95% CI)	*P*-value
T2DM patient	Non-diabetic patient	2.9 (1.1 - 7.5)	0.025	1.3 (0.4 - 3.7)	0.661
Age (years)		1.0 (1.0 - 1.1)	0.056		
Male	Female	1.6 (0.7 - 3.7)	0.266		
Stage 3-4 disease	Stage 1-2 disease	0.7 (0.3 - 1.8)	0.462		
Squamous cell carcinoma	Adenocarcinoma	4.0 (1.7 - 9.5)	0.002	2.1 (0.8 - 5.6)	0.128
Number of tumor lesions ≥ 1 mL per patient		0.8 (0.7 - 0.9)	0.001	0.8 (0.7 - 0.9)	0.004
MTV_patient_ > median	MTV_patient_ < median	0.5 (0.2 - 1.1)	0.091		

Only patients with adenocarcinoma and squamous cell carcinoma histology were included in this analysis (*N*=96). 95% CI, 95% confidence interval; MTV_patient_, sum of the metabolic tumor volume measured in all tumor lesions per patient; T2DM, type 2 diabetes mellitus; OR, odds ratio.

## Abbreviations

2-DG: 2-deoxyglucose
95% CI: 95% confidence interval
CT: computed tomography
EARL: European Association of Nuclear Medicine Research Ltd
^18^F-FDG: 2-deoxy-2-[fluorine-18]fluoro-D-glucose
GLUT-1: glucose transporter 1
MTV: metabolic tumor volume
MTVpatient: sum of the metabolic tumor volume measured in all tumor lesions per patient
NSCLC: non-small cell lung cancer
OR: odds ratio
PET: positron emission tomography
PVE: partial volume effect
ROI: region of interest
SUVmax: maximum measured standardized uptake value
SUVmean: mean standardized uptake value
T1DM: type 1 diabetes mellitus
T2DM: type 2 diabetes mellitus
