# Supplementary material for: 18F-FDG PET/CT Scans Can Identify Sub-Groups of NSCLC Patients with High Glucose Uptake in the Majority of Their Tumor Lesions
Source: J Cancer. 2021 Jan 1;12(2):562–70. doi: 10.7150/jca.45899 (PMC7738988; doi:10.7150/jca.45899)
Supplement: Supplementary file 1 — Supplementary figures. [file jcav12p0562s1.pdf]

Supplemental methods

The ROIs were manually drawn around each visible ^{18}F-FDG avid tumor lesion on the 3D images in AMIDE. Symbia.net and Syngo.via software (Siemens) with a better display resolution were used to confirm that all visible tumor lesions were found using AMIDE. The AMIDE software provides the ^{18}F-FDG uptake in Becquerel per cubic cm (Bq/cc) and the associated volume in cubic mm (mm^3). The SUV and MTV were calculated using standard formulas in Excel 2007 software. SUV = (0.001 x measured uptake (Bq/cc)) / (radioactivity at the moment of scanning (MBq) / patient's weight (kg)) [24]. Radioactivity at the moment of scanning (MBq) = injected dosage (MBq) x (0.5)^(time after injection (min) / half-life (min)). For ^{18}F-FDG a half-life time of 109.771 min was used. The SUVmean 40%, corrected for the serum blood glucose levels according to the EARL standards, was used for all tumor lesion analyses (SUVcorrected = SUV x (fasting serum glucose (mmol/L) / 5)) [24]. MTV (mL) = measured volume (mm^3) / 1000. For these calculations, it was assumed that one cc is equivalent to 1 g. The patient's weight in kg, the injected dosage of ^{18}F-FDG in MBq and the time between the moment of ^{18}F-FDG administration and the moment of scanning in min, were used as stated in the patient records.

Supplemental Figures

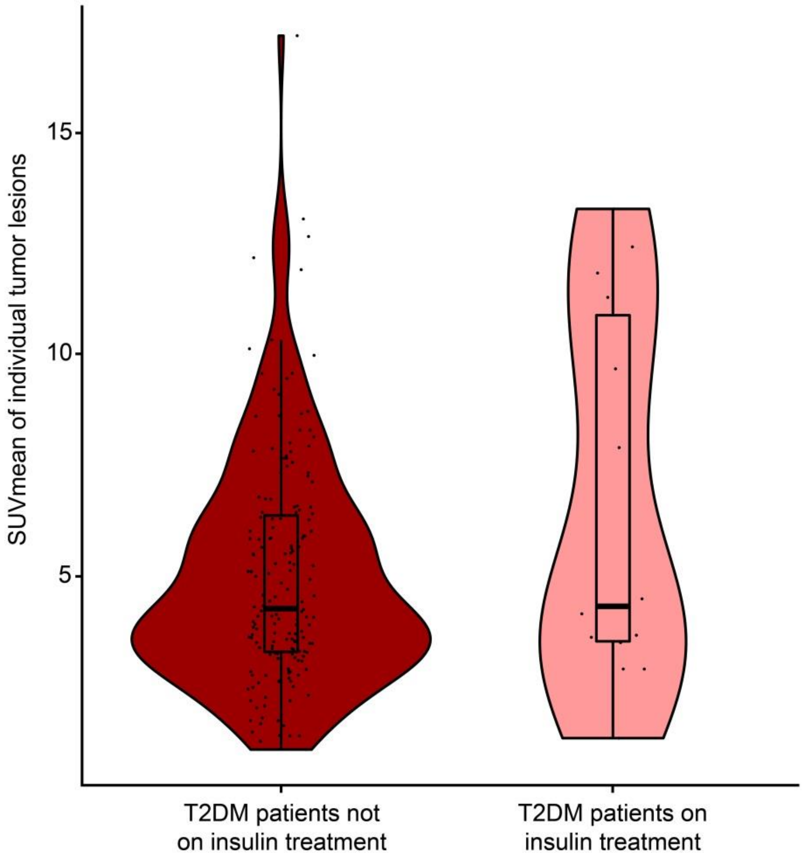

Figure S1: Insulin treatment did not influence the distribution of ^{18}F-FDG tumour lesion uptake in type 2 diabetes mellitus (T2DM) patients compared to T2DM patients not on insulin treatment. Violin plots of all mean standardized uptake values (SUVmean) measured in all tumor lesions with a volume ≥1 mL visible on the primary diagnostic ^{18}F-FDG PET/CT scans of non-small cell lung cancer (NSCLC) patients with concurrent type 2 diabetes mellitus (T2DM). Of these patients, 14 were on insulin treatment and 175 were not on insulin treatment. Box plots show the median (horizontal bar), the first and third quartile. Dots are representing individual NSCLC tumor lesions.

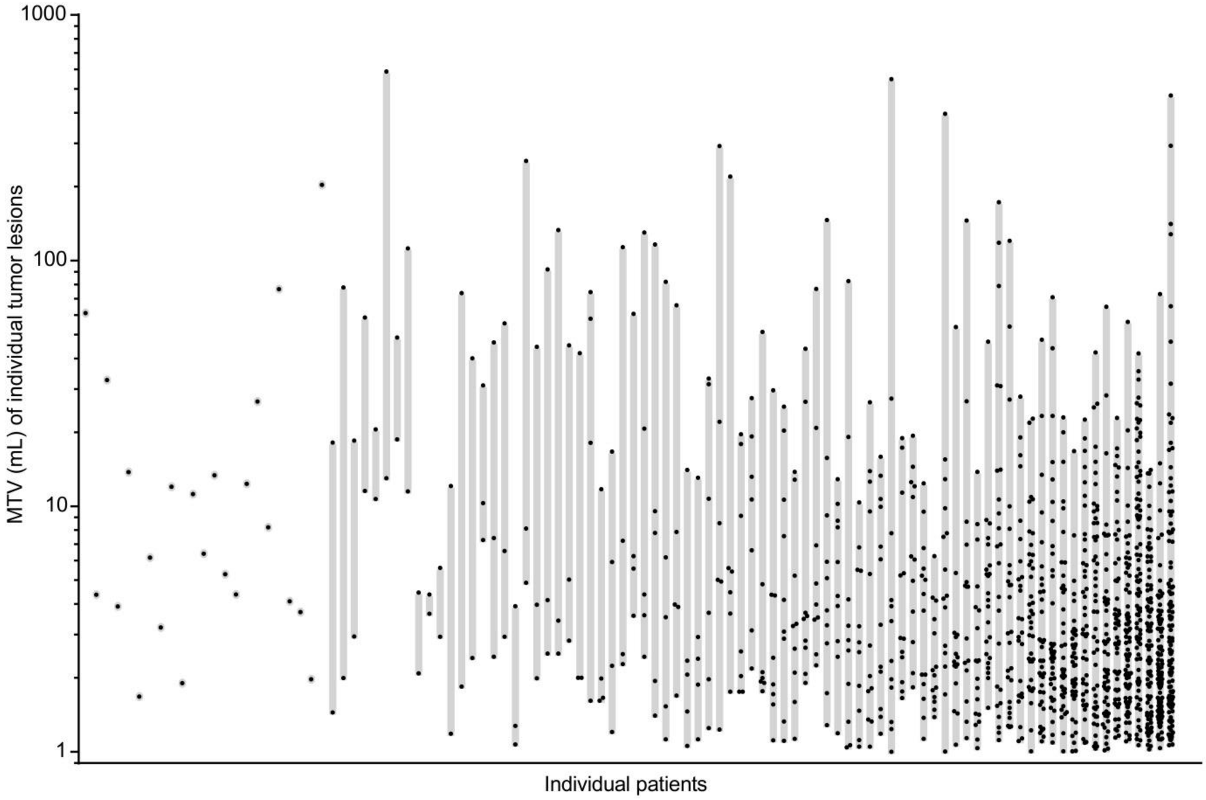

Figure S2: Patients with higher number of tumor lesions more frequently had smaller tumor lesions. Each grey bar represents an individual patient. Each black dot represents a single tumor lesion with a metabolic tumor volume (MTV) ≥1 mL (*N*=1394). The length of the grey bars represents the range between the lowest and highest MTV of all tumor lesions per patient. Patients were ordered on the x-axis based on number of tumor lesions (low to high).

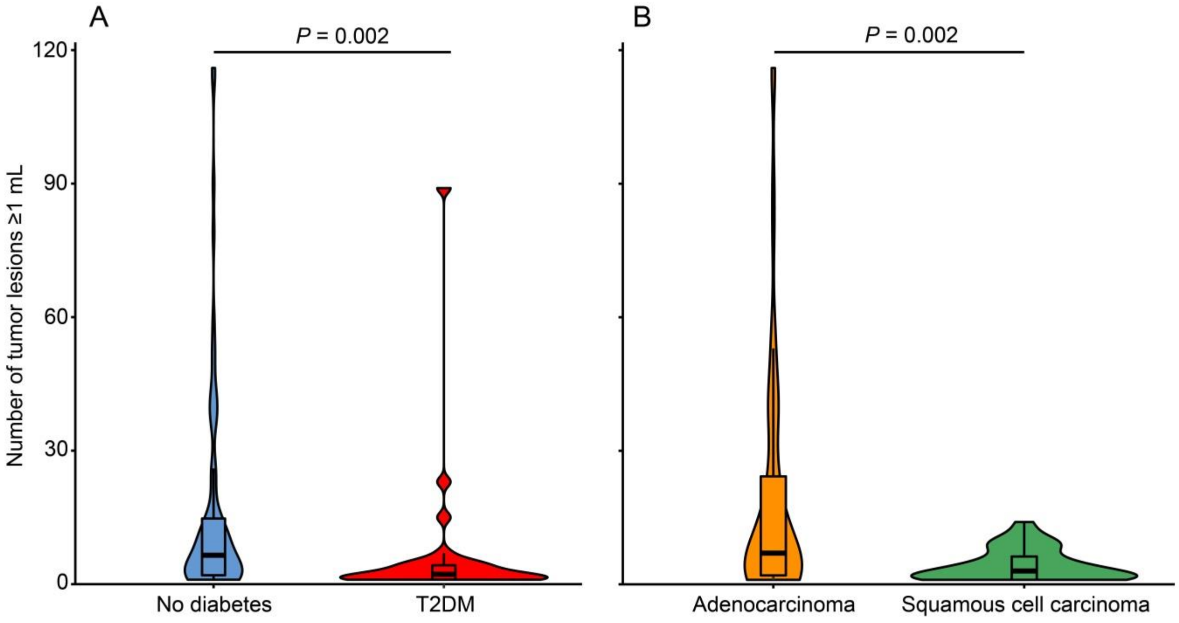

Figure S3: Non-small cell lung cancer (NSCLC) patients with type 2 diabetes mellitus (T2DM) or squamous cell carcinoma histology more often had low number of tumor lesions per patient. A) This figure shows the number of tumor lesions ≥1 mL found per patient stratified for non-diabetic (*N*=74) and diabetic (*N*=28) NSCLC patients. **B)** This figure shows the number of tumor lesions ≥1 mL per patient stratified for patients with adenocarcinoma (*N*=60) and patients with squamous cell carcinoma (*N*=36). Box plots showing the median (horizontal bar), the first and third quartile. Dots are representing individual NSCLC tumor lesions.
